# Predictors of interferon-gamma release assay results and their association with COVID-19 infection outcomes

**DOI:** 10.5588/ijtldopen.24.0180

**Published:** 2024-10-01

**Authors:** S.J.W. Kang, G.W. Eather, F. Qureshi, J.R. Scott

**Affiliations:** ^1^ Department of Respiratory, Sleep and Mycobacterial Medicine, Princess Alexandra Hospital, Metro South Health, Brisbane, QLD, Australia;; ^2^ Metro South Clinical Tuberculosis Service, Princess Alexandra Hospital, Metro South Health, Brisbane, QLD, Australia;; ^3^ Faculty of Medicine, The University of Queensland, Brisbane, QLD, Australia;; ^4^ QCIF Bioinformatics, Institute for Molecular Bioscience, The University of Queensland, Brisbane, QLD, Australia.

**Keywords:** IGRA, Coronavirus disease 2019, immunosuppression

## Abstract

**BACKGROUND:**

An ‘indeterminate’ interferon-gamma release assay (IGRA) result used in the diagnosis of latent TB infection (LTBI) is most commonly due to an inadequate control (or ‘mitogen’) response, which may reflect underlying T-cell dysfunction.

**METHODS:**

We performed a single-centre, retrospective study on COVID-19 patients admitted to a tertiary referral hospital who had IGRA testing conducted over a 5-month period. The primary outcomes included predictors of indeterminate IGRA results and associations with COVID-19 outcomes.

**RESULTS:**

A total of 181 patients were included for analysis. Approximately one-third of patients hospitalised with COVID-19 with IGRA testing performed (60/181) had an indeterminate result. The likelihood of an indeterminate IGRA was increased in patients with a history of solid organ transplant and a higher severity of COVID-19 at the time of testing. An indeterminate IGRA was associated with a higher risk of severe COVID-19 and a higher risk of admission to the ICU during admission to the hospital. No difference in mortality between the two subgroups was found.

**CONCLUSION:**

Our study demonstrated that COVID-19 patients on immunosuppression had a high likelihood of an indeterminate IGRA result, which was associated with markers of disease severity and immunosuppression. In this cohort, an indeterminate result was associated with worse COVID-19 outcomes.

## RÉSUMÉ

**CONTEXTE:**

Un résultat « indéterminé » du test de libération d'interféron-gamma (IGRA, pour l’anglais « *interferon-gamma release assay* »), employé pour diagnostiquer l'infection tuberculeuse latente (LTBI pour l’anglais « *latent TB infection* »), est généralement attribué à une réponse de contrôle insuffisante (ou « mitogène »), ce qui peut indiquer un dysfonctionnement sous-jacent des cellules T.

**MÉTHODES:**

Nous avons mené une étude rétrospective unicentrique portant sur des patients atteints de COVID-19, admis dans un hôpital de référence de niveau tertiaire, ayant subi un test IGRA sur une durée de 5 mois. Les résultats principaux ont mis en évidence les facteurs prédictifs des résultats IGRA indéterminés ainsi que les corrélations avec les résultats liés au COVID-19.

**RÉSULTATS:**

Au total, 181 patients ont été inclus dans l'analyse. Environ un tiers des patients hospitalisés pour COVID-19 ayant subi un test IGRA (60 sur 181) ont présenté un résultat indéterminé. La probabilité d'obtenir un résultat IGRA indéterminé était plus élevée chez les patients ayant des antécédents de greffe d'organe solide et ceux présentant une sévérité accrue de la maladie au moment du test. Un résultat IGRA indéterminé était corrélé à un risque accru de COVID-19 sévère ainsi qu'à une probabilité plus élevée d'admission en unité de soins intensifs lors de l'hospitalisation. Aucune différence significative de mortalité n'a été observée entre les deux sous-groupes.

**CONCLUSION:**

Notre recherche a révélé que les patients souffrant de COVID-19 et recevant un traitement immunosuppresseur présentaient une probabilité élevée d'obtenir un résultat IGRA indéterminé, ce qui était corrélé avec des indicateurs de gravité de la maladie et d'immunosuppression. Au sein de cette cohorte, un résultat indéterminé était lié à des issues moins favorables dans le contexte de l'étude sur le COVID-19.

The interferon-gamma release assay (IGRA) is used to identify immune correlates of previous exposure to *Mycobacterium tuberculosis* and forms the basis of the diagnosis of latent TB infection (LTBI). This is performed on whole blood and relies on an enzyme-linked immunosorbent assay to quantify interferon-gamma (IFN-γ) liberated after exposure of T-cells to *M. tuberculosis*-specific antigens. Two control tubes are utilised, one measuring background levels of IFN-γ and a second measuring the response to a non-specific T-cell stimulant (the mitogen tube). The mitogen response is a marker of the integrity of the immune response that helps validate a negative result. An inadequate mitogen response is one of the common causes of an ‘indeterminate’ result, particularly in immune-suppressed patients, which reflects a deficient T-cell response.^1^

Rates of indeterminate IGRA results have been demonstrated to be between 2% and 11%, with higher rates in populations with chronic conditions such as rheumatologic disease or immunosuppressive states.^2,3^ A low lymphocyte count is a common correlate of an indeterminate IGRA due to inadequate mitogen control.^3,4^

Coronavirus disease 2019 (COVID-19), a cause of significant morbidity and mortality worldwide, is known to induce a pro-inflammatory state with concomitant T-cell dysfunction.^5^ This reduces the ability to produce IFN-γ, which is required in determining and interpreting IGRA test results.^6^ Furthermore, it has been documented that COVID-19 can cause immune dysregulation in patients, resulting in cytokine storms and diffuse damage to organs, especially pulmonary parenchyma. This may also result in systemic hyperinflammatory and pro-coagulative state.^7,8^ The use of anti-inflammatory and immunosuppressive medications such as corticosteroids and baracitinib (a Janus kinase [JAK] inhibitor) reduces the effects of innate immune response to COVID-19 on organs.^9,10^

We evaluated a cohort of patients hospitalised with COVID-19 who had IGRA testing as part of a screening protocol for opportunistic infections on commencement of corticosteroids. IGRA testing was included due to the potential risk of LTBI reactivation.^11^ Given the relationship between IGRA results and immune dysfunction in this cohort, we performed a retrospective analysis to explore the predictors of an indeterminate IGRA result and outcomes in hospitalised patients with COVID-19.

## METHODS

We performed a single-centre retrospective study involving all patients admitted for COVID-19 and had IGRA testing performed during their admission to a tertiary referral hospital over a 6-month period from 1 October 2021 to 31 March 2022. QuantiFERON-TB Gold Plus^®^ (Qiagen, Hilden, Germany) was employed for IGRA testing on commencement of corticosteroids (daily 6 mg dexamethasone orally or 8 mg dexamethasone intravenously), according to local COVID-19 protocols. The results of IGRA testing were interpreted according to the manufacturer’s criteria. The primary endpoints investigated in this study were the predictors of IGRA results in hospitalised patients with COVID-19 and the association between IGRA results and COVID-19-related outcomes.

Ethics approval was provided by the Metro South Health Human Research Ethics Committee, Metro South Health, Brisbane, QLD, Australia (HREC/2022/QMS/86309).

The inclusion criteria for our study included all consecutive patients admitted during the study period with COVID-19 infection who had IGRA testing performed during their hospital admission. Exclusion criteria included any patients below the age of 18, patients who did not receive any form of immunosuppression during their hospital admission, and patients with a previous diagnosis of active TB or LTBI.

Data were collected on patient demographics, baseline immunosuppression, determinants of IGRA testing results and COVID-19-related outcomes. High-risk countries of birth were defined as countries with a TB incidence of 40 cases per 100,000 persons or greater. Fully vaccinated patients were defined as those who had received at least two doses of an Australian government-approved COVID-19 vaccination at the time of the study. COVID-19 severity at the time of testing was determined by the need for supplemental oxygen therapy and/or high-dependency unit (HDU) or intensive care unit (ICU) admission, with a ‘low COVID-19 severity’ defined as no requirement for either supplemental oxygen therapy or HDU/ICU admission, and a ‘high COVID-19 severity’ defined as a requirement for supplemental oxygen therapy and/or HDU/ICU admission. Baseline immunosuppressive therapy included any form of immunosuppressive medication, such as immunomodulator/disease-modifying therapy for rheumatological conditions or chemotherapeutic agents. Markers of infection, such as neutrophil count, lymphocyte count and C-reactive protein at the time of COVID-19 diagnosis, were recorded.

COVID-19-related outcomes that were recorded in our study included the highest recorded COVID-19 severity in the hospital, the highest level of respiratory support received during the participant’s hospital admission, the need for ICU/HDU admission, length of stay and mortality in the hospital.

Statistical analysis was performed using R v4.0.2 (R Computing, Vienna, Austria). Categorical variables were described using frequencies and percentages. Continuous variables were described using median and interquartile range (IQR). Univariate logistic regressions were fitted for analyses investigating associations with IGRA results and COVID-19 outcomes. All the variables for predictors influencing IGRA test results and COVID-19 outcomes were also evaluated in a multivariate model to account for potential confounding between variables. Variables with *P* < 0.2 in the univariate analysis were identified as candidates for the multivariate model and remained in the final multivariate model if *P* < 0.05. A combination of backwards step-wise elimination and manual elimination was used to derive the final multivariate models.

## RESULTS

Of the 181 patients included in our study, 117 had a negative IGRA result, while 60 had an indeterminate result. All indeterminate IGRA results were due to an inadequate mitogen response, interpreted according to the manufacturer’s criteria. The baseline demographics of patients in the study are summarised in Table 1. Four patients with positive IGRA results were excluded from further analysis due to a small sample size. Age, sex, country of birth, body mass index and vaccination status were similar across the IGRA-negative and -indeterminate groups. The proportion of patients with chronic obstructive pulmonary disease (COPD) and hypertension was higher in the IGRA-negative group. There was a higher proportion of patients with a history of solid organ transplant (SOT) in the IGRA-indeterminate group.

**Table 1.** Demographics of study participants by IGRA subgroup.

Demographic	IGRA result		
	Negative	Indeterminate	Positive
	(*n* = 117)	(*n* = 60)	(*n* = 4)
	*n* (%)	*n* (%)	*n* (%)
Age, years, median [IQR]	66 [56–76]	65.5 [53.75–73]	68 [57–79]
Male sex	74 (63)	36 (60)	2 (50)
Country of birth (high-risk)	13 (11)	9 (15)	3 (75)
BMI, kg/m^2^, median [IQR]	29.8 [24.9–36.2]	28.69 [24.0–33.7]	27.4 [23.1–30.9]
Fully vaccinated	70 (60)	33 (50)	1 (25)
COPD	27 (23)	7 (12)	0 (0)
Hypertension	63 (53)	20 (33)	3 (75)
Diabetes mellitus	37 (32)	21 (35)	3 (75)
History of malignancy (excluding skin)	17 (15)	12 (20)	1 (25)
History of solid organ transplant	3 (3)	8 (13)	0 (0)
Connective tissue disease	4 (3)	3 (5)	0 (0)
Autoimmune disease	15 (13)	7 (12)	0 (0)

IGRA = interferon-gamma release assay; IQR = interquartile range; BMI = body mass index; COPD = chronic obstructive pulmonary disease.

Table 2 outlines predictors of IGRA results. Patients on immunosuppressive medications at baseline were likelier to test IGRA-indeterminate (odds ratio [OR] 2.33, 95% confidence interval [CI] 1.15–4.73). This was most evident in SOT recipients (OR 5.85, 95% CI 1.62–27.50). An IGRA-indeterminate result was also more likely in patients with a higher COVID-19 severity at the time of testing (OR 2.52, 95% CI 1.32–4.92) and in those whose IGRA test was performed ≥2 days after commencement of corticosteroids (OR 3.11, 95% CI 1.36–7.27). A patient’s vaccination status did not affect IGRA results. A higher C-reactive protein (CRP) count at the time of testing was associated with a higher likelihood of an indeterminate IGRA result, while neutrophil and lymphocyte counts did not affect IGRA results.

**Table 2.** Predictors of IGRA results.

Predictor	IGRA result		OR (95% CI)	*P*-value
	Negative	Indeterminate		
	(*n* = 117)	(*n* = 60)		
	*n* (%)	*n* (%)		
High-risk country of birth	13 (11)	9 (15)	1.41 (0.43–3.49)	0.5
COPD	27 (23)	7 (12)	0.44 (0.17–1.03)	0.07
Hypertension	63 (53)	20 (33)	0.43 (0.22–0.81)*	0.01*
Diabetes mellitus	37 (32)	21 (35)	1.16 (0.60–2.24)	0.7
History of malignancy (excluding skin)	17 (15)	12 (20)	1.47 (0.64–3.31)	0.4
History of solid organ transplant	3 (3)	8 (13)	5.85 (1.62–27.50)*	0.011*
Connective tissue disease	4 (3)	3 (5)	1.49 (0.28–6.96)	0.6
Autoimmune disease	15 (13)	7 (12)	0.90 (0.33–2.27)	0.8
Baseline immunosuppressive therapy			2.33 (1.15–4.73)*	0.019*
No	95 (81)	39 (65)		
Yes	22 (19)	21 (35)		
Fully vaccinated			0.82 (0.44–1.54)	0.5
No	47 (40)	27 (45)		
Yes	70 (60)	33 (55)		
COVID-19 severity at the time of testing			2.52 (1.32–4.92)*	0.006*
Low	63 (54)	19 (32)		
High	54 (46)	41 (68)		
Timing of IGRA test				
Before corticosteroids	55 (47)	21 (35)		
1 day after corticosteroids	46 (39)	20 (33)	1.14 (0.55–2.36)	0.7
≥2 days after corticosteroids	16 (14)	19 (32)	3.11 (1.36–7.27)*	0.008*
Lymphocyte count	1.0 (0.6–1.4)	0.8 (0.5–1.2)	0.86 (0.58–1.02)	0.3
Neutrophil count	5.0 (3.3–6.6)	6.1 (4.2–9.1)	1.01 (0.97–1.07)	0.5
C-reactive protein	46 (20–108)	72 (37–145)	1.01 (1.00–1.01)*	0.023*

* Statistically significant.

IGRA = interferon-gamma release assay; OR = odds ratio; CI = confidence interval; COPD = chronic obstructive pulmonary disease; COVID-19 = coronavirus disease 2019.

As outlined in Table 3, an indeterminate IGRA result predicted a longer median length of hospital stay (OR 1.08, 95% CI 1.03–1.14) and was associated with worse patient-related outcomes, including an increased risk of high-severity COVID-19 infection in hospital (OR 2.52, 95% CI 1.26–5.30), a higher likelihood of requiring non-invasive ventilation (OR 6.04, 95% CI 2.12–18.60), invasive ventilation (OR 3.4, 95% CI 1.0–11.5), and HDU (OR 2.88, 95% CI 1.21–6.88) or ICU admission (OR 4.30, 95% CI 1.90–10.00). There was no significant association between IGRA results and in-patient mortality.

**Table 3.** COVID-19-related outcomes.

COVID-19 related outcome	IGRA result		OR (95% CI)	*P*-value
	Negative	Indeterminate		
	(*n* = 117)	(*n* = 60)		
	*n* (%)	*n* (%)		
Length of stay, days, median [IQR]	6 [4–9]	9 [6–13]	1.08 (1.03–1.14)*	0.002*
Highest level of COVID-19 severity during admission			2.52 (1.26–5.30)*	0.012*
Low	48 (41%)	13 (22%)		
High	69 (59%)	47 (78%)		
Highest level of respiratory support				
Room air	34 (29%)	9 (15%)	—	
Low-flow nasal oxygen	48 (41%)	17 (28%)	1.34 (0.54–3.47)	0.5
High-flow nasal oxygen	16 (14%)	10 (17%)	2.36 (0.80–7.09)	0.12
Non-invasive ventilation	10 (9%)	16 (27%)	6.04 (2.12–18.60)*	0.001*
Invasive ventilation	9 (8%)	8 (13%)	3.36 (1.01–11.50)*	0.048*
Highest level of care received				
General ward	90 (77%)	29 (48%)	—	
High dependency unit	14 (12%)	13 (22%)	2.88 (1.21–6.88)*	0.016*
Intensive care unit	13 (11%)	18 (30%)	4.30 (1.90–10.00)*	<0.001*
Mortality	12 (10%)	5 (8%)	0.82 (0.25–2.31)	0.6

* Statistically significant.

COVID-19 = coronavirus disease 2019; IGRA = interferon-gamma release assay; OR = odds ratio; CI = confidence interval.

Multivariate analysis of predictors of IGRA results (Table 4), initially using all the variables from Table 2 with *P* < 0.2, revealed that a higher COVID-19 severity at the time of testing (OR 2.81, 95% CI 1.36–6.09) and history of SOT (OR 19.2, 95% CI 3.98–146) were both independently associated with an indeterminate IGRA result. A multivariate analysis of variables and potential confounders affecting individual COVID-19 outcomes during admission (Table 5) showed that an indeterminate IGRA result was independently associated with a higher risk of high-severity COVID-19 infection in hospital (OR 2.85, 95% CI 1.32–6.49) and a higher likelihood of HDU/ICU admission in hospital (OR 2.62, 95% CI 1.25–5.59).

**Table 4.** Multivariate logistic regression for predictors of indeterminate IGRA results.

Predictor	OR (95% CI)	*P*-value
Hypertension	0.34 (0.16–0.71)	0.005
COVID-19 severity at the time of testing	2.81 (1.36–6.09)	0.007
History of solid organ transplant	19.2 (3.98–146)	<0.001

IGRA = interferon-gamma release assay; OR = odds ratio; CI = confidence interval; COVID-19 = coronavirus disease 2019.

**Table 5.** Multivariate logistic regression for COVID-19-related outcomes.

COVID-19-related outcome	Risk factor	OR (95% CI)	*P*-value
Length of stay			
	Age	1.03 (1.00–1.05)	0.025
	High initial COVID-19 severity	3.56 (1.85–7.12)	<0.001
	History of solid organ transplant	3.92 (1.06–16.6)	0.047
	Autoimmune disease	3.86 (1.44–11.4)	0.010
High COVID-19 severity during admission			
	Indeterminate IGRA result	2.85 (1.32–6.49)	0.010
	Timing of IGRA test		
	1 day after corticosteroids	5.73 (2.63–13.30)	<0.001
	≥2 days after corticosteroids	3.14 (1.26–8.37)	0.017
Supplemental oxygen or respiratory support requirement			
	Obesity	2.49 (1.13–571)	0.026
	Timing of IGRA test		
	1 day after corticosteroids	9.23 (3.49–29.5)	<0.001
	≥2 days after corticosteroids	4.18 (1.56–2.18)	0.007
HDU or ICU requirement			
	Indeterminate IGRA result	2.62 (1.25–5.59)	0.011
	High initial COVID-19 severity	3.98 (1.86–9.04)	<0.001
	History of malignancy	0.30 (0.09–0.87)	0.037

COVID-19 = coronavirus disease 2019; OR = odds ratio; CI = confidence interval; IGRA = interferon-gamma release assay; HDU = high dependency unit; ICU = intensive care unit.

## DISCUSSION

Our study demonstrates several predictors of an indeterminate IGRA, which had associations with COVID-19 outcomes in hospitalised patients. The rate of an indeterminate IGRA in our cohort was approximately 33%, which is significantly higher than figures in previous studies in Australia and worldwide.^12,13^ This likely reflects a different population where patients admitted with COVID-19 were more likely to have a dysfunctional immune system, either from premorbid immunosuppression in some patients that potentially necessitated their admission or from the direct effects of COVID-19 infection itself with the associated dysfunctional immune response.^3^

Although local protocols requested that all hospitalised patients have IGRA testing performed before the commencement of any form of immunosuppression (including corticosteroids), our study showed that a large proportion of patients had testing performed after the commencement of corticosteroids, which had an impact on IGRA test results. This could have been due to several factors, including patient acuity on presentation requiring urgent commencement of treatment, unavailability of pathology/phlebotomy staff, and lack of awareness of guidelines among non-COVID-19-specific ward staff.

Several predictors of an indeterminate IGRA result were demonstrated in our study. Previous studies have determined that underlying immunosuppression resulted in a higher likelihood of an indeterminate IGRA result, reflected in our cohort of patients.^2,14^ This was especially evident in patients who were SOT recipients who required management with multiple immunosuppressive agents to reduce the risk of organ rejection. Background immunosuppression downregulates the type 1 T-helper pathway responsible for generating IFN-γ directed towards pathogens such as fungi and mycobacteria, which is directly assessed with the mitogen control tube in IGRA testing.^15,16^ This would also explain the higher likelihood of obtaining an indeterminate IGRA result when testing was performed after immunosuppression was commenced for COVID-19, which was noted in our study.

Furthermore, our study has demonstrated that higher severity of COVID at the time of testing also impacted IGRA testing results. Previous studies of the immune response to COVID-19 have emphasised that CD4, CD8 and lymphocyte depletion are associated with more severe COVID-19 infections.^17^ Possible explanations for the lymphocyte depletion seen with the disease include sequestration of cytokines and immune cells from blood into the lungs, T-cell exhaustion and viral-induced lymphocyte destruction.^17,18^

Given the main reason for performing IGRA testing before commencing immunosuppression in our cohort was to identify patients with LTB at risk of reactivation and require treatment, this study raises the question of the utility of performing non-targeted testing for patients with COVID-19 (and other diseases affecting immunity) given the high proportion of indeterminate results. Targeted testing at patients with a high pre-test probability of LTBI and ensuring testing is performed before the commencement of immunosuppression or immunomodulatory treatment may improve the yield and accuracy of testing. This study also emphasises the need for better LTBI diagnostic tests, less affected by a patient’s immune function or concurrent illnesses.

Several previous studies have examined the relationship between IGRA results and COVID-19 outcomes. Torre et al. evaluated 335 patients with severe COVID-19 who had IGRA testing performed with the commencement of immunosuppression and found that the mortality rate was significantly higher in patients who had an indeterminate IGRA compared to other IGRA results.^3^ Solanich et al. studied 96 patients admitted for COVID-19 and found that indeterminate results were significantly more likely in patients with more severe COVID-19 infections.^19^ In contrast, Ward et al. found no significant difference in COVID severity or mortality when comparing negative and indeterminate IGRA results in a cohort of hospitalised patients.^20^

Our study demonstrates that an indeterminate IGRA predicted several adverse outcomes in patients hospitalised with COVID-19, such as a higher risk of severe COVID-19 infection and a higher likelihood of HDU/ICU admission. This supports the findings of previous studies that suggested that an indeterminate IGRA result is a marker of higher COVID-19 disease severity, presumably related to the degree of T-cell dysfunction from pre-existing immune system deficits or COVID-19 infection itself.^3,20^ The utility of IGRA testing as a marker for COVID-19 severity remains undetermined. While it may potentially have some value as a surrogate marker of immune dysfunction, there are other more easily accessible and interpretable biomarkers which have been shown to correlate with COVID-19 infection outcomes.^21^

Some of the limitations of this study include being a retrospective study and therefore being subject to bias, including selection bias, where patients with more severe COVID-19 were more likely to have screening for opportunistic infections performed during their hospital admission. Length of stay in hospital may have been influenced by other factors rather than due to the disease itself, such as other comorbidities or social reasons.

Another major limitation of this study was the lack of follow-up data and repeated testing for indeterminate IGRA results. Although not one of the outcomes of this study, it is worth noting that none of those with indeterminate IGRA results were followed up by the hospital clinicians specifically for repeat testing and management after discharge. Given the uncertainty associated with an indeterminate result, these patients should have had follow-up testing after recovering from their acute illness.

The lack of this strategy being included in the protocol reflected the escalating crisis that marked this pandemic, with protocols hastily designed to ensure all potential adverse outcomes were captured but without evidence to guide the implementation of such protocols. Our cohort of patients was referred to their general practitioners for follow-up testing after hospital discharge, but no formal process was in place to ensure this follow-up occurred.

## CONCLUSION

In conclusion, our study demonstrates that an indeterminate IGRA was associated with markers of disease severity and immunosuppression in patients hospitalised with COVID-19. An indeterminate result was also associated with worse COVID-19 outcomes in this cohort. This result highlights the importance of recognising patients with impaired immunity and a potential need for using markers of immunity for prognostication and determining management in COVID-19 and other acute diseases. The utility of performing IGRA as a test for detecting LTBI in immunosuppressed individuals is limited due to the high rate of indeterminate results, and patients should perhaps be stratified based on the pre-test probability of LTBI. Targeted retesting of individuals with an indeterminate result after a period of recovery may help determine patients at risk of TB reactivation and warrants further investigation.
